# Machine Learning for Predicting Distant Metastasis of Medullary Thyroid Carcinoma Using the SEER Database

**DOI:** 10.1155/2023/9965578

**Published:** 2023-12-30

**Authors:** Zhen-Tian Guo, Kun Tian, Xi-Yuan Xie, Yu-Hang Zhang, De-Bao Fang

**Affiliations:** ^1^Department of General Surgery, Beijing Electric Power Hospital, State Grid Corporation China, Capital Medical University, Beijing 100073, China; ^2^Fujian Provincial Hospital, Fuzhou, Fujian 350001, China; ^3^Mudanjiang Medical University, Mudanjiang, Heilongjiang 157000, China; ^4^Hefei National Laboratory for Physical Sciences at Microscale, School of Basic Medical Sciences, Division of Life Sciences and Medicine, University of Science and Technology of China, Hefei 230027, Anhui, China

## Abstract

**Objectives:**

We aimed to establish an effective machine learning (ML) model for predicting the risk of distant metastasis (DM) in medullary thyroid carcinoma (MTC).

**Methods:**

Demographic data of MTC patients were extracted from the Surveillance, Epidemiology, and End Results (SEER) database of the National Institutes of Health between 2004 and 2015 to develop six ML algorithm models. Models were evaluated based on accuracy, precision, recall rate, *F*1-score, and area under the receiver operating characteristic curve (AUC). The association between clinicopathological characteristics and target variables was interpreted. Analyses were performed using traditional logistic regression (LR).

**Results:**

In total, 2049 patients were included and 138 developed DM. Multivariable LR showed that age, sex, tumor size, extrathyroidal extension, and lymph node metastasis were predictive features for DM in MTC. Among the six ML models, the random forest (RF) had the best predictability in assessing the risk of DM in MTC, with an accuracy, precision, recall rate, *F*1-score, and AUC higher than those of the traditional binary LR model.

**Conclusion:**

RF was superior to traditional LR in predicting the risk of DM in MTC and can provide a valuable reference for clinicians in decision-making.

## 1. Introduction

As a result of changes in living environments, heightened health awareness, and advances in detection technology, the incidence of thyroid cancer has experienced a considerable increase in most parts of the world [1]. Medullary thyroid carcinoma (MTC) is a relatively rare malignancy, constituting approximately 5% of all thyroid malignancies. Patients with MTC generally exhibit a poorer prognosis than those with differentiated thyroid cancer (DTC), with MTC accounting for approximately 13% of all thyroid cancer-related fatalities [2, 3]. Roughly 75% of MTC cases are sporadic, while around 25% are autosomal dominant [4]. Research has demonstrated that mutations in *RET*, a proto-oncogene, are present in approximately 6% of sporadic MTC patients and up to 98% of familial-inherited MTC patients [5]. Studies have indicated that extrathyroidal extension and distant metastasis (DM) are significant predictors of poor prognosis in patients [6, 7]. At the time of initial diagnosis, 10%–15% of MTC patients present with DM [8]. DM of MTC may involve the bones, lungs, and liver [9]. The American Thyroid Association's guidelines for the management of medullary thyroid cancer recommend various imaging examinations for MTC, potentially involving DM, including enhanced CT, MRI, abdominal ultrasound, and bone scans [10]. These diagnostic methods have a sensitivity of approximately 50%–80% for metastatic diseases. In recent years, the clinical application of drugs targeting *RET* proto-oncogene mutations has been proven to be effective in treating MTC patients with *RET* mutations [11]. Consequently, early diagnosis of MTC with DM and early intervention for high-risk patients may significantly improve patient survival.

Machine learning (ML) is a subfield of artificial intelligence technology. Compared to traditional predictive models, ML can enhance the accuracy of models by uncovering nonlinear relationships in large datasets [12, 13]. During medical treatment, vast amounts of data from patients are generated. Therefore, processing and analyzing these data using ML can offer a reliable reference for clinicians to diagnose diseases and prognosticate outcomes. Thus, our study aimed to develop a model based on the Surveillance, Epidemiology, and End Results (SEER) database to predict the occurrence of DM in patients with MTC.

## 2. Materials and Methods

### 2.1. Data Sources and Study Population

Data for this study were acquired from the SEER public databases, utilizing SEER^*∗*^Stat 8.4.0.1 software for data extraction. Our study focused on patients diagnosed with MTC in the United States between 2004 and 2015. We excluded patients with missing data, unclear clinical and pathological conditions, uncertain histological classifications, or other types of thyroid cancer (TC). The histological types were restricted to medullary carcinomas. According to the International Classification of Diseases (ICD) for Oncology-3, patients' histological codes are 8345/3 and 8510/3, adopting AJCC 7th edition TNM stage. Variables included age, sex (male or female), race (White, Black, and others), year of diagnosis, Spanish-Hispanic origin, laterality (unilateral and bilateral), multifocality (solitary and multifocal), tumor size, extrathyroidal extension, lymph node metastasis, MTC subtypes, and DM. Distant metastasis means that the tumor invades at least one or more target organs such as brain, bone, liver, lung, and so on. As the SEER database contains public data, informed consent from relevant patients for the use of the SEER database for research purposes was not required, nor was the ethical approval. Our request for access to the SEER data was approved by the National Cancer Institute, USA (reference number 19238-Nov2021).

### 2.2. Screening for Risk Factors and Model Construction

Statistical analysis was conducted using SPSS software (version 26.0; IBM Corporation). In the univariable analysis, we employed Pearson's correlation analysis to examine the association between predictor variables, with results being presented in the form of heat maps. The predictive factors related to DM were initially screened through univariable analysis (*p* < 0.05), and the variables that met the criteria were incorporated into a multivariable logistic regression (LR) analysis. The receiver operating characteristic (ROC) curve was plotted and analyzed based on the results. An area under the ROC curve (AUC) greater than 0.5 was considered meaningful. All computed *p* values were two-sided, and statistical significance was accepted at <0.05.

**Figure 1:**
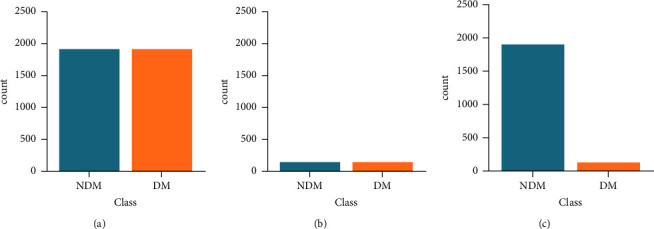
The distribution of the target variables after the sampling process. (a) Oversampling data, (b) undersampling data, and (c) target variable distribution of original data.

**Figure 2:**
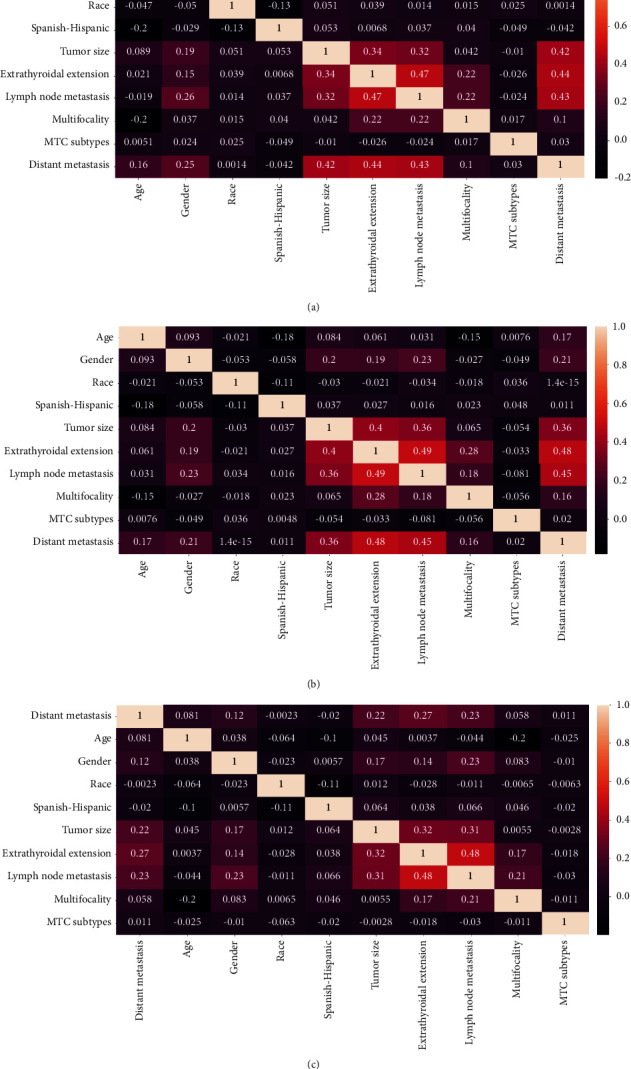
Heatmaps of the correlation between characteristic features of the patients in different datasets. (a) Oversampling data, (b) undersampling data, and (c) original data.

The rate of DM of patients with MTC in the SEER database was low, resulting in an unbalanced original dataset. To establish a more accurate prediction model, it is essential to address this imbalance. In this study, we employed two techniques for processing the original dataset: oversampling and undersampling. We then used a correlation matrix to analyze the original and processed data. The synthetic minority oversampling technique (SMOTE) and undersampling are standard approaches for balancing class distribution in imbalanced datasets, widely used to improve prediction models [14]. The distribution of the target variables after the sampling process is illustrated in Figure 1. After data processing, the correlation between variables became more apparent, as demonstrated in Figure 2.

We used Python software (version 3.9.12, Python Software Foundation) to incorporate the selected variables include all variables in the ML model and construct a prediction model. The technically processed data (oversampled and undersampled data) were randomly divided into a training set (80%) and a test set (20%). The training set employed six commonly used ML algorithms: decision tree (DT), support vector machine (SVM), random forest (RF), k-nearest neighbors (KNN), extreme gradient boosting (XGBoost), and gradient boosting machine (GBM). Model evaluation was primarily based on accuracy, precision, recall, *F*1-score, and AUC value. The model with the highest AUC value was selected as the optimal model.

## 3. Results

### 3.1. Analysis of Patient Information

This study included a total of 2049 MTC patients, of which 138 (6.7%) developed DM and the remaining 1911 (93.3%) did not. The baseline characteristics of all patients are presented in Table 1.

In the univariable LR analysis, DM was significantly associated with age, sex, multifocality, tumor size, extrathyroidal extension, and lymph node metastasis (*p* < 0.05) (Table 2). These characteristic variables were incorporated into the multivariable LR analysis.

**Figure 3:**
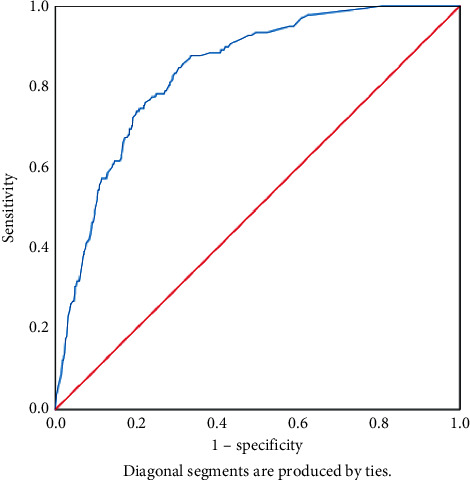
LR models predict the ROC curve of distant metastasis in MTC patients.

In the multivariable LR analysis, age [15] sex, extrathyroidal extension, lymph node metastasis, and tumor size were identified as independent predictors of DM in MTC. However, multifocality was not an independent predictive factor for the occurrence of DM in MTC. Further details can be found in Table 2. The ROC curve was plotted based on traditional multivariable LR results (AUC = 0.838, 95% confidence interval (CI): 0.808–0.868, *p* < 0.001). Detailed information is summarized in Figure 3.

**Figure 4:**
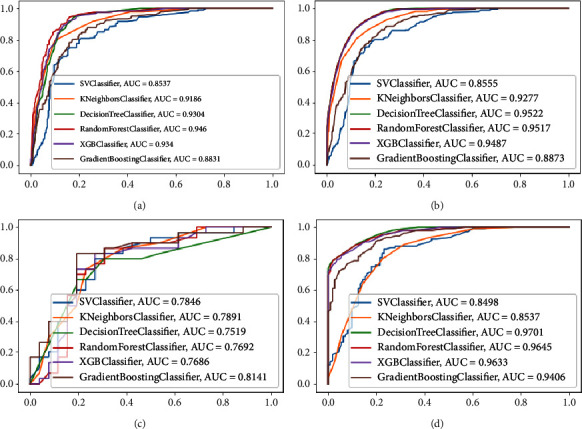
ROC curves of six ML algorithms in different datasets. (a) The ROC curves of the six ML algorithms model in the test set with oversampling. (b) The ROC curves of the six ML algorithms model in the training set with oversampling. (c) The ROC curves of the six ML algorithms model in the test set with undersampling. (d) The ROC curves of the six ML algorithms model in the training set with undersampling. ROC, receiver operating characteristic; ML, machine learning; AUC, area under the receiver operating characteristic curve.

**Figure 5:**
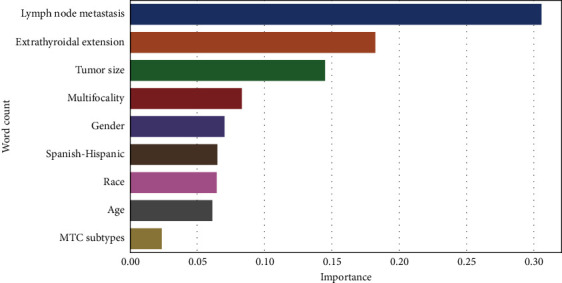
Feature importance derived from the RF model. The plot shows the relative importance of the variables in the RF model. MTC, medullary thyroid carcinoma.

For the analysis of the ML algorithm, six ML models were constructed and evaluated based on accuracy, precision, recall rate, *F*1-score, and AUC value. It was observed that ML models constructed after data oversampling outperformed those constructed after undersampling. Tables 3 and 4 provide details on the six ML models constructed from the over- and undersampled data. The ROC curves of the six ML models, constructed by oversampling and undersampling in the training and test sets, are depicted in Figure 4. In the models established using oversampled data, the AUC of all models was greater than 0.850, with the RF model performing better than the other models. The RF model demonstrated accuracy, precision, recall rate, *F*1-score, and AUC value of 0.890, 0.847,0.946, 0.894, and 0.946, respectively, as well as a higher AUC value than the LR model. This indicates that the diagnostic efficiency of the ML algorithm surpasses that of the traditional LR model and exhibits excellent prediction performance. Employing RF for feature selection, as illustrated in Figure 5, revealed that lymph node metastasis was the most critical factor in determining whether MTC patients also have DM.

This study developed an online network calculator for evaluating the risk of distant metastasis in MTC patients, which can be applied to clinical patients (https://121.43.117.60:8000/).

## 4. Discussion

Patients with MTC account for only 5% of the total number of individuals newly diagnosed with TC, while the global incidence rate of MTC is rising rapidly. Deaths from MTC comprise approximately 13% of the total mortality rate of TC, and the 10-year overall survival rate of MTC ranges between 65% and 71%. However, when MTC occurs with DM, the 10-year overall survival rate can decrease to 40–44% [15, 16]. MTC neither concentrates radioactive iodine nor is it inhibited by thyroxine [17]. Total thyroidectomy is the primary treatment method for MTC, with the decision to perform lymph node dissection depending on the specific situation. Adjuvant radiation therapy can be considered for MTC patients with incomplete resection, a high risk of local recurrence, or DM [10]. Radiotherapy can provide continuous control in patients with DM and prevent further progression [18]. However, the impact of radiotherapy on patients' survival rates remains controversial. In patients without DM, radiotherapy may cause more harm than good [19]. Some perspectives suggest that the role of radiation therapy in MTC is limited to patients who are ineligible or have contraindications for surgical treatment or targeted drugs [20]. Targeted drugs are recommended for patients with DM, particularly because studies have demonstrated [11, 21] that *RET*-specific inhibitors (selpercatinib and pralsetinib) are effective and promising therapies for MTC patients with DM and progression. The prognosis and treatment effectiveness of MTC are largely related to tumor staging; therefore, early diagnosis is a crucial objective in the management of MTC patients [22]. Previous research on MTC has mostly focused on prognosis and analysis of survival [23, 24].

However, there are few studies on the DM of MTC. Utilizing independent predictors to predict DM can help physicians better evaluate patients with MTC and provide them with more effective individualized treatment options.

Univariable analysis showed that age, sex, multifocality, tumor size, extrathyroidal extension, and lymph node metastasis were independent predictors of DM. However, multivariable analysis indicated that multifocality could not serve as an independent predictor of DM in patients with MTC. This finding is consistent with the conclusion of the RF feature selection, and it is generally believed that multilocality has an independent predictive effect on cervical lymph node metastasis in MTC [25]. Nonetheless, multifocality had a relatively small impact on predicting the occurrence of DM in patients with MTC, which aligns with findings of previous research [25, 26]. RF feature selection revealed that extrathyroidal extension was a key factor in predicting DM, while lymph node metastasis was the most important predictor of DM, consistent with a previous study [26]. We also identified tumor size was an important predictor. Compared with tumors larger than 4 cm, the odds ratio (OR) for tumors of 2–4 cm and ≤2 cm was 0.555 and 0.287, respectively. As tumor size gradually increases, the risk of DM in MTC also increases. Tumor size significantly impacts the recurrence and long-term survival rates of MTC [24]. Extrathyroidal extension and tumor size are also crucial predictive factors for lymph node and DM in MTC [6, 16]. Meanwhile, extrathyroidal extension and tumor size are directly related to T staging in TNM staging, suggesting that tumor stage can also serve as a predictive factor for DM. Contrary to a previous study [27], sex was considered as an independent predictor of DM. We also discovered that female sex was a protective factor for DM. This conclusion is similar to that of a previous study [26]. In our study, 55 years of age was used as the cutoff age [27] and it showed that older patients were more likely to develop DM than younger patients. Therefore, older patients should be actively followed up and regularly examined. In this study, race could not independently predict DM in patients with MTC, which is consistent with results of previous research [26, 27]. In traditional LR, MTC subtypes and Spanish-Hispanic could not be used as independent predictors, and their influence on the feature selection of RF was also small.

We constructed six predictive models based on the SEER database to predict DM in patients with MTC and evaluated six algorithmic models based on accuracy, precision, recall rate, *F*1-score, and AUC value. We employed the SMOTE technique to address unbalanced datasets and concluded that, for unbalanced datasets used to build ML models, SOMTE is superior to undersampling [14]. By oversampling and undersampling, we enhanced the performance of the model and determined that the prediction model established by oversampling outperformed the one established by undersampling. This may be attributed to fewer patients with DM among MTC patients, resulting in limited ability of the model to identify key predictive factors for patients with combined DM. This study established six ML algorithms, among which RF demonstrated excellent predictive performance (AUC = 0.946), surpassing that of the traditional LR model (AUC = 0.838). Therefore, RF was the best model for predicting MTC patients with DM using the SEER database.

## 5. Limitations

However, there are some limitations to this study. First, as this study is based on demographics of North American, other populations should be used for validation in future research. Second, the predictive performance of the model warrants further optimization, and additional predictive factors potentially related to DM should be incorporated into the prediction model in future studies. Finally, due to the limitations of the database, tumor markers such as CEA and AFP were not included in MTC patients. We will continue to improve and supplement the model in future studies.

## 6. Conclusions

In conclusion, this study aimed to identify independent predictors of DM in patients with MTC and to develop a prediction model utilizing ML algorithms. Our analysis, based on the SEER database, demonstrated that age, sex, tumor size, extrathyroidal extension, and lymph node metastasis were significant independent predictors of DM in MTC patients. The RF ML algorithm outperformed the traditional LR model in predicting DM, providing a more accurate and reliable tool for clinical use.

The application of the SMOTE technique for addressing unbalanced datasets was proven to be effective in enhancing the performance of the prediction model. Our findings underscore the importance of early diagnosis and individualized treatment plans for MTC patients, ultimately contributing to improved patient outcomes.

## Figures and Tables

**Table 1:** The detailed demographic information of the patients with MTC.

Categories	With DM (*n* = 138)	Without DM (*n* = 1911)	*p* value
Age, *n* (%)			<0.001
<55	47 (34.1%)	959 (50.2%)	
≥55	91 (65.9%)	952 (49.8%)	
Gender (*n*%)			<0.001
Female	50 (36.2%)	1149 (60.1%)	
Male	88 (63.8%)	762 (39.9%)	
Race (*n*%)			0.891
White	116 (84.1%)	1619 (84.7%)	
Black	14 (10.1%)	159 (8.3%)	
Other	8 (5.8%)	133 (7.0%)	
Year of diagnosis			0.367
2004–2009	54 (39.1%)	823 (43.1%)	
2010–2015	84 (60.9%)	1088 (56.9%)	
Spanish-Hispanic-Latino (*n*%)			0.372
Yes	17 (12.3%)	289 (15.1%)	
No	121 (87.7%)	1622 (84.9%)	
MTC subtypes (*n*%)			0.622
MTC with amyloid stroma	4 (2.9%)	71 (3.7%)	
MTC NOS^a^	134 (97.1%)	1840 (93.2%)	
Laterality (*n*%)			0.419
Unilateral	138 (100%)	1902 (99.5%)	
Bilateral	0 (0%)	9 (0.5%)	
Multifocality (*n*%)			0.009
Solitary tumor	86 (62.3%)	1388 (72.6%)	
Multifocal tumor	52 (37.7%)	523 (37.7%)	
Tumor size (*n*%)			<0.001
≤2	27 (19.6%)	1030 (53.9%)	
2–4	46 (33.3%)	594 (31.1%)	
≥4	65 (47.1%)	287 (15.0%)	
Extrathyroidal extension (*n*%)			<0.001
Yes	57 (41.3%)	309 (16.2%)	
No	81 (58.7%)	1602 (83.8%)	
Lymph node metastasis (*n*%)			<0.001
No	24 (17.4%)	1206 (63.1%)	
Cervical central lymph node	36 (26.1%)	288 (15.1%)	
Cervical lateral lymph node	70 (50.7%)	359 (18.8%)	
Yes NOS	8 (5.8%)	58 (3.0%)	

MTC, medullary thyroid carcinoma; DM, distant metastasis; NOS, not otherwise specified.

**Table 2:** Univariable analysis and multivariable analysis of variables related to distant metastasis.

	Univariable analysis			Multivariable analysis		
	OR	95% CI	*p* value	OR	95% CI	*p* value
Age (year)						
<55	0.513	0.357–0.513	<0.001	0.480	0.323–0.713	<0.001
≥55	Ref			Ref		
Gender						
Female	0.377	0.263–0.540	<0.001	0.664	0.450–0.980	0.039
Male	Ref			Ref		
Race						
White	1.191	0.569–2.491	0.642			
Black	1.464	0.596–3.596	0.406			
Other	Ref					
Spanish-Hispanic						
Yes	Ref					
No	1.268	0.752–2.139	0.373			
MTC subtypes						
MTC with amyloid stroma	0.774	0.278–2.150	0.623			
MTC NOS	Ref					
Multifocality						
Solitary tumor	0.623	0.435–0.892	0.010	0.866	0.578–1.298	0.486
Multifocal tumor	Ref			Ref		
Tumor size (cm)						
≤2	0.116	0.073–0.185	<0.001	0.287	0.173–0.476	<0.001
2–4	0.342	0.229–0.512	<0.001	0.555	0.360−0.0855	0.008
≥4	Ref			Ref		
Extrathyroidal extension						
Yes	Ref			Ref		
No	0.136	0.095–0.195	<0.001	0.364	0.240–0.554	<0.001
LNM						
No	0.144	0.062–0.335	<0.001	0.327	0.132–0.806	0.015
Cervical central lymph node	0.906	0.401–2.050	0.813	1.021	0.437–2.385	0.962
Cervical lateral lymph node	1.414	0.647–3.091	0.386	1.269	0.564–2.858	0.565
Yes NOS	Ref			Ref		

MTC, medullary thyroid carcinoma; NOS, not otherwise specified; OR, odds ratio; CI, confidence interval.

**Table 3:** Comparison of prediction performance between different models constructed from oversampling data.

Model	Accuracy	AUC	Precision	Recall rate	*F*1-score
DT	0.764	0.930	0.782	0.724	0.752
RF	0.890	0.946	0.847	0.946	0.894
SVC	0.781	0.853	0.761	0.811	0.785
KNN	0.830	0.918	0.777	0.917	0.836
GBM	0.813	0.883	0.788	0.848	0.817
XGBoost	0.879	0.934	0.851	0.915	0.882

AUC, area under the receiver operating characteristic curve; DT, decision tree; SVM, support vector machine; RF, random forest; KNN, k-nearest neighbors; XGBoost, extreme gradient boosting; GBM, gradient boosting machine.

**Table 4:** Comparison of prediction performance between different models constructed from undersampling data.

Model	Accuracy	AUC	Precision	Recall rate	*F*1-score
DT	0.803	0.751	0.827	0.800	0.813
RF	0.732	0.769	0.741	0.766	0.754
SVC	0.750	0.784	0.750	0.800	0.774
KNN	0.714	0.789	0.750	0.700	0.760
GBM	0.785	0.814	0.781	0.833	0.806
XGBoost	0.767	0.768	0.774	0.800	0.786

AUC, are under the receiver operating characteristic curve; DT, decision tree; SVM, support vector machine; RF, random forest; KNN, k-nearest neighbors; XGBoost, extreme gradient boosting; GBM, gradient boosting machine.

## Data Availability

The dataset presented in this study can be found at https://seer.cancer.gov. Further inquiries can be directed to the corresponding author.
